# Relationship between METS-IR and thyroid cancer incidence in Korea: a nationwide population-based study

**DOI:** 10.3389/fonc.2024.1383864

**Published:** 2024-04-11

**Authors:** Hye Ryeon Kim, Minkook Son, Seok Jae Huh, Sang Yi Moon, Hyeyeon Moon, Yeo Wool Kang, Myeongseok Koh, Jong Yoon Lee

**Affiliations:** ^1^ Department of Internal Medicine, Dong-A University College of Medicine, Busan, Republic of Korea; ^2^ Department of Physiology, Dong-A University College of Medicine, Busan, Republic of Korea; ^3^ Department of Data Sciences Convergence, Dong-A University Interdisciplinary Program, Busan, Republic of Korea; ^4^ Department of Internal Medicine, Yeongdo Hospital, Busan, Republic of Korea

**Keywords:** thyroid cancer, incidence, metabolic syndrome, insulin resistance, METS-IR

## Abstract

**Background:**

Several previous studies found a positive relationship between metabolic syndrome (MetS) and thyroid cancer (TC) risk. However, there is no research that has studied the relationship between the metabolic score for insulin resistance (METS-IR), a novel surrogate marker for IR, and TC incidence. Thus, we designed this retrospective cohort study to evaluate the relationship between the incidence of TC and METS-IR.

**Method:**

We analyzed a cohort of 314,321 Korean adults aged over 40 years who participated in the National Health Screening Program from 2009 to 2010. The individuals were divided into four groups based on METS-IR quartiles. Follow-up was until the diagnosis of TC or death, or until December 31, 2019, if neither. The relationship between METS-IR and TC incidence was analyzed using the Cox proportional-hazards model with multi-variable adjustments.

**Results:**

A total of 4,137 participants (1.3%) were diagnosed with TC during a mean follow-up of 9.5 ± 1.5 years. The population with Q1 METS-IR scores showed higher disease-free probabilities than those with Q4 METS-IR scores (*p <*0.001). The hazard ratio (95% confidential interval) for TC incidence in Q2, Q3, and Q4 METS-IR value were 1.14 (1.05 to 1.25), 1.21 (1.11 to 1.33), and 1.30 (1.18 to 1.42) compared with Q1 of METS-IR, respectively. The incidence of TC tended to increase with increasing METS-IR values in the total population, especially the male population in the restricted cubic spline. In subgroup analysis, the TC risk was more pronounced in the subgroups under 65 and with a BMI < 25 kg/m^2^.

**Conclusion:**

METS-IR was positively correlated with TC incidence in Korea.

## Introduction

Thyroid cancer (TC) is the third most common cancer, following lung and stomach cancer, and the highest age-standardized incidence rate in Korea in 2020 (1). In women, it has the second highest crude and age-standardized incidence rates following breast cancer (1). In the past several decades, the incidence of TC has increased rapidly, in part due to improved detection technology and generalized fine-needle aspiration biopsies (2, 3). However, other risk factors for the development of TC cannot be ignored (4).

Metabolic syndrome (MetS) includes a cluster of metabolic disorders, including overweight, dysglycemia, hypertension, and dyslipidemia, and is significantly associated with an increased risk of developing diabetes mellitus and cardiovascular disease (5–7). Insulin resistance (IR) is known as one of the major mechanisms of MetS (7). Several models, such as hyperinsulinemic-euglycemic glucose clamp technique, homeostatic model assessment (HOMA), has been developed for determining insulin sensitivity or resistance (8, 9). However, these methods have some problems, including that is labor- and time-intensive or clinically less useful (8, 9). Thus, with the increasing interest for IR assessment in clinical settings, easier and more clinically practical markers have been in need. In 2018, the novel score, metabolic score for IR (METS-IR) was developed, which was calculated based on fasting blood glucose (FBG) levels, fasting triglyceride (TG) levels, body mass index (BMI), and high-density lipoprotein cholesterol (HDL-C) levels (10). As METS-IR demonstrated the better diagnostic and/or predicting performance to determine MetS compared to other makers, it has been used as surrogate marker for IR, which is valuable in predicting the development of MetS (10–13).

IR was reported to impact the incidence of several solid tumors, including colorectal, lung, and pancreatic cancer (14–17). A previous study reported that thyroid volumes were larger, and the risk of thyroid nodules was higher in IR patients (18). The relationship between MetS and TC has been suggested in several studies (19, 20), and it was also reported in a systemic review and meta-analysis (21). However, the study did not use METS-IR as a predictive value for TC and had a limitation due to differences in IR definitions between the studies analyzed (21).

The aim of this large retrospective cohort study was to evaluate the relationship between METS-IR, a novel score for insulin sensitivity, and the incidence of TC, especially in Korean individuals who participated in the National Health Screening Program (NHSP).

## Methods

### Study design and population

This was a retrospective population-based cohort study that used claims data obtained from the National Health Insurance Service-National Health Screening Cohort (NHIS-HealS) in Korea. The NHIS is the sole government insurer, providing a mandatory universal insurance system for the Korean population. It maintains a database of demographics, inpatient and outpatient claims, medical procedures, prescription drugs, and diagnoses according to the International Classification of Diseases, 10th edition (ICD-10). Additionally, the NHSP is provided to all insured individuals aged over 40 years every two years. This program includes anthropometric measurements, laboratory test results, and self-reported questionnaires about lifestyle behaviors. The NHIS-HealS database was established using data from NHSP participants.

**Figure 1:**
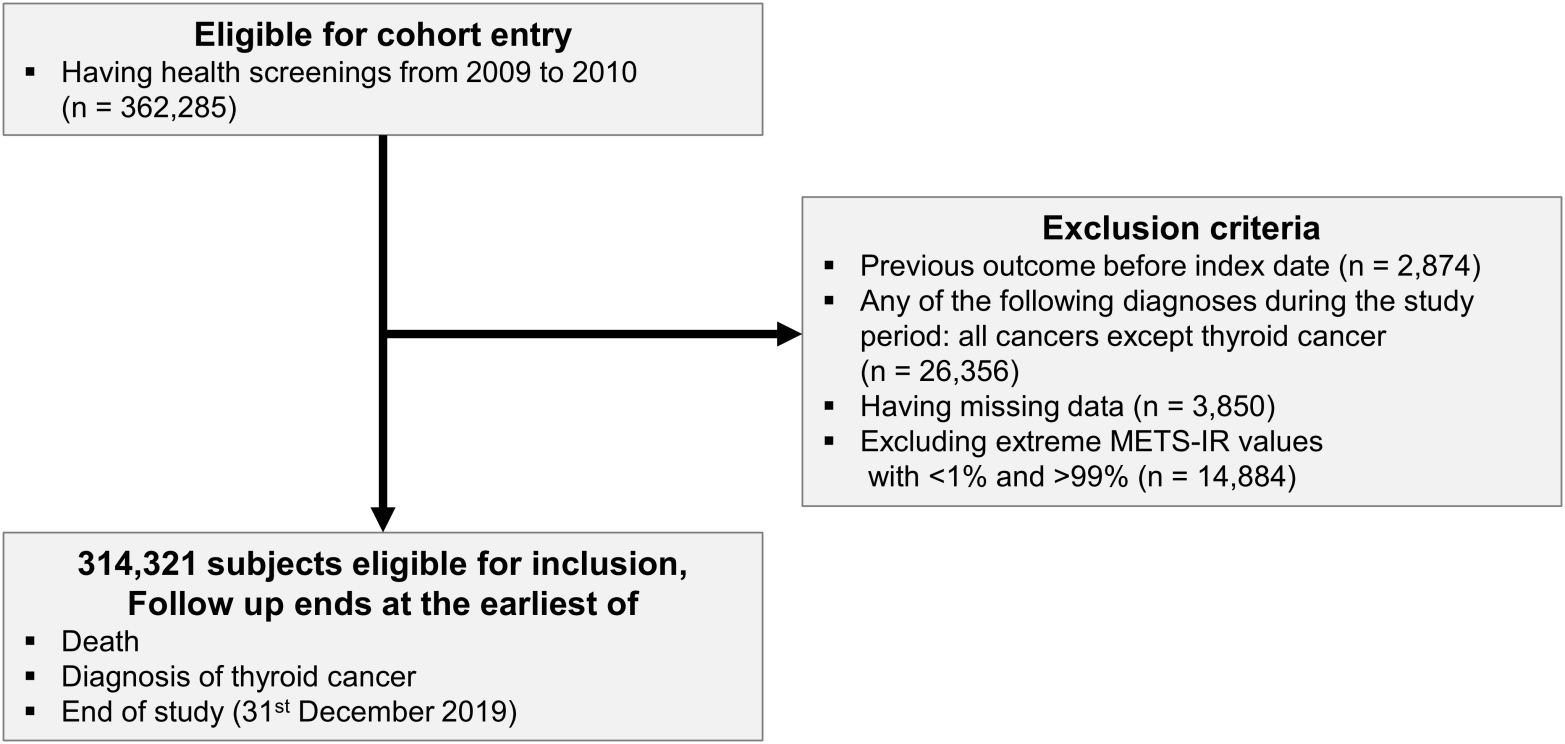
Flow of the study population.

We analyzed a cohort of Korean adults aged over 40 years (n = 362,285) who participated in the NHSP from 2009 to 2010, utilizing data from the NHIS-HealS database. The index date was set to the NHSP. We excluded individuals from this cohort who had TC before the index date (n = 2,874) and individuals who were diagnosed with cancers other than TC during the follow-up period (n = 26,356). We also excluded subjects with missing data (n = 3,850) and subjects with extreme METS-IR values (< 1% or > 99%) (n = 14,884). Finally, 314,321 subjects were analyzed in this study. Follow-up was until TC diagnosis or death, or until December 31, 2019, if neither (**Figure 1**).

### Definitions of key variables

METS-IR was defined as ln [(2 × FBG (mg/dL) + serum TG level (mg/dL)) × BMI (kg/m^2^)]/ln [serum HDL-C level (mg/dL)]. After removing extreme METS-IR values (< 1 or > 99 percentile), the participants were divided into four groups based on METS-IR quartiles (Q1, Q2, Q3, and Q4). We utilized NHIS data to identify individuals newly diagnosed with TC for data analysis with a primary focus on insurance claims. Patients with TC were identified based on the ICD-10 diagnostic code C73 (**Supplementary Table 1**).

### Definitions of covariates

Income level was categorized into quantiles, and residence was divided into urban and rural. The definitions of hypertension, diabetes, and dyslipidemia are described in **Supplementary Table 1**. The Charlson comorbidity index (CCI), a method of predicting mortality by classifying or weighting comorbid conditions, was calculated using diagnoses within one year before index data in the claims data (22). BMI was calculated as body weight in kilograms divided by height in meters squared (kg/m^2^). Obesity was defined as a BMI of ≥ 25 kg/m^2^ according to the guidelines provided by the World Health Organization considering the Asia-Pacific perspective. The laboratory results included hemoglobin levels (mg/dL) and estimated glomerular filtration rate (eGFR) (ml/min/1.73m^2^). Self-reported questionnaires were used to collect data on current smoking, alcohol drinking, and regular exercise habits.

### Statistics and data analysis

Variables are reported as means with frequencies and percentages or standard deviations. Disease-free probability between the METS-IR and TC was visualized using a Kaplan–Meier curve and evaluated using log-rank test. The Cox proportional hazards model was used to evaluate the risk of TC with adjustments for multiple covariates, including sex, age, income level, residence, underlying disease, and health screening items. However, we adjusted the model for METS-IR for the aforementioned factors, excluding BMI. Hazard ratios (HRs) and the corresponding 95% confidence intervals (CIs) were used to determine the association between surrogate markers of IR and TC. The Schoenfeld residuals test with the logarithm of the cumulative hazard function based on Kaplan-Meier estimates was used to evaluate proportional hazard assumptions. No disturbance to the assumption that risk was proportional over time were seen. We also employed restricted cubic splines to examine HR trends along with their 95% CIs for TC, according to METS-IR. We used four knots at the 5th, 35th, 65th, and 95th percentiles according to METS-IR values, and the mean METS-IR values were selected as reference values in the restricted cubic spline. For additional sensitivity analysis, we used 1:1 propensity score matching in comparing Q1 and Q4. Matching variables included sex, age, income level, residence, underlying diseases including CCI, and health screening items including smoking, alcohol drinking, and exercise status. Propensity score matching was performed using the nearest neighbor matching method, and the caliper was set to less than 0.01. Statistical analyses were performed using SAS (version 9.4, SAS Institute Inc., Cary, NC, USA) and R 3.6.0 (R Foundation for Statistical Computing, Vienna, Austria) to ensure the accuracy and reliability of the results. *P*-values of less than 0.05 were considered statistically significant.

### Ethics statement

The study protocol was thoroughly reviewed and approved by the Institutional Review Board of Dong-A University Hospital (DAUHIRB-EXP-23-177), and the study was conducted in accordance with the Declaration of Helsinki. As this study used data from the National Health Insurance Service-National Health Screening Cohort in Korea, formal consent was not required.

## Results

### Baseline characteristics of the study population

The baseline characteristics of the study population, including demographics, underlying disease, and health screening, and quartiles based on METS-IR values are described in **Table 1**. The baseline characteristics of men and women are described in **Supplementary Tables 2** and **3**, respectively. The mean METS-IR values for the Q1 and Q4 are 2.1 and 2.5, respectively.

**Table 1:** Baseline characteristics of the study population according to METS-IR values.

All subjects (N=314,321)	METS-IR				P-value
	1^st^ quartile, Q1 (N=78,595)	2^nd^ quartile, Q2 (N=78,624)	3^rd^ quartile, Q3 (N=78,517)	4^th^ quartile, Q4 (N=78,585)	
Demographics					
Age (years)	58.5 (9.1)	58.5 (8.6)	58.9 (8.5)	59.3 (8.6)	< 0.001
Sex (%)					< 0.001
Male	42348 (53.9)	42395 (53.9)	42277 (53.8)	42380 (53.9)	
Female	36247 (46.1)	36229 (46.1)	36240 (46.2)	36205 (46.1)	
Income level (%)					< 0.001
1^st^ quartile	11287 (14.4)	10906 (13.9)	10813 (13.8)	10948 (13.9)	
2^nd^ quartile	17392 (22.1)	16254 (20.7)	15603 (19.9)	15605 (19.9)	
3^rd^ quartile	22562 (28.7)	22810 (29.0)	23239 (29.6)	24036 (30.6)	
4^th^ quartile	27354 (34.8)	28654 (36.4)	28862 (36.8)	27996 (35.6)	
Residence (%)					< 0.001
Urban	51744 (65.8)	51072 (65.0)	50516 (64.3)	49097 (62.5)	
Rural	26851 (34.2)	27552 (35.0)	28001 (35.7)	29488 (37.5)	
Underlying disease					
Hypertension (%)	25974 (33.0)	33291 (42.3)	39377 (50.2)	47761 (60.8)	< 0.001
Diabetes (%)	5014 (6.4)	8270 (10.5)	11949 (15.2)	18112 (23.0)	< 0.001
Dyslipidemia (%)	18388 (23.4)	25762 (32.8)	33441 (42.6)	46142 (58.7)	< 0.001
Charlson comorbidity index					< 0.001
0	41402 (52.7)	37933 (48.2)	35094 (44.7)	31238 (39.8)	
1	21109 (26.9)	21772 (27.7)	21846 (27.8)	21461 (27.3)	
2	9268 (11.8)	10303 (13.1)	11091 (14.1)	12229 (15.6)	
≥ 3	6816 (8.7)	8616 (11.0)	10486 (13.4)	13657 (17.4)	
Health screening					
Body mass index (kg/m^2^)	21.0 (1.5)	23.2 (1.4)	24.8 (1.5)	27.1 (2.1)	< 0.001
Systolic blood pressure (mmHg)	121.6 (15.3)	124.4 (15.0)	126.4 (14.8)	128.8 (14.8)	< 0.001
Diastolic blood pressure (mmHg)	75.5 (9.8)	77.1 (9.8)	78.2 (9.8)	79.6 (9.8)	< 0.001
Fasting blood glucose (mg/dL)	94.3 (16.3)	98.1 (19.5)	101.6 (22.5)	107.5 (28.0)	< 0.001
Total cholesterol (mg/dL)	198.2 (35.3)	200.6 (36.8)	201.6 (37.9)	200.9 (38.4)	< 0.001
Triglycerides (mg/dL)	94.6 (42.3)	117.7 (53.5)	142.5 (65.5)	181.1 (80.0)	< 0.001
HDL cholesterol (mg/dL)	63.3 (16.3)	55.7 (11.1)	50.9 (10.3)	45.3 (9.6)	< 0.001
LDL cholesterol (mg/dL)	116.2 (35.3)	121.4 (35.7)	122.3 (37.3)	119.6 (37.8)	< 0.001
Hemoglobin (g/dL)	13.6 (1.4)	13.8 (1.5)	13.9 (1.5)	14.0 (1.5)	< 0.001
Glomerular filtration rate (mL/min/ 1.73 m^2^)	80.4 (30.3)	79.0 (30.2)	77.9 (31.4)	76.8 (31.7)	< 0.001
Current smoker (%)	14793 (18.8)	12573 (16.0)	12411 (15.8)	12977 (16.5)	< 0.001
Drink alcohol (%)	31921 (40.6)	32026 (40.7)	31227 (39.8)	29875 (38.0)	< 0.001
Exercise regularly (%)	3749 (4.8)	3829 (4.9)	3641 (4.6)	3357 (4.3)	< 0.001
METS-IR	28.7 (2.0)	33.3 (1.3)	36.9 (1.4)	42.5 (2.9)	< 0.001

### Association between METS-IR and thyroid cancer incidence

**Figure 2:**
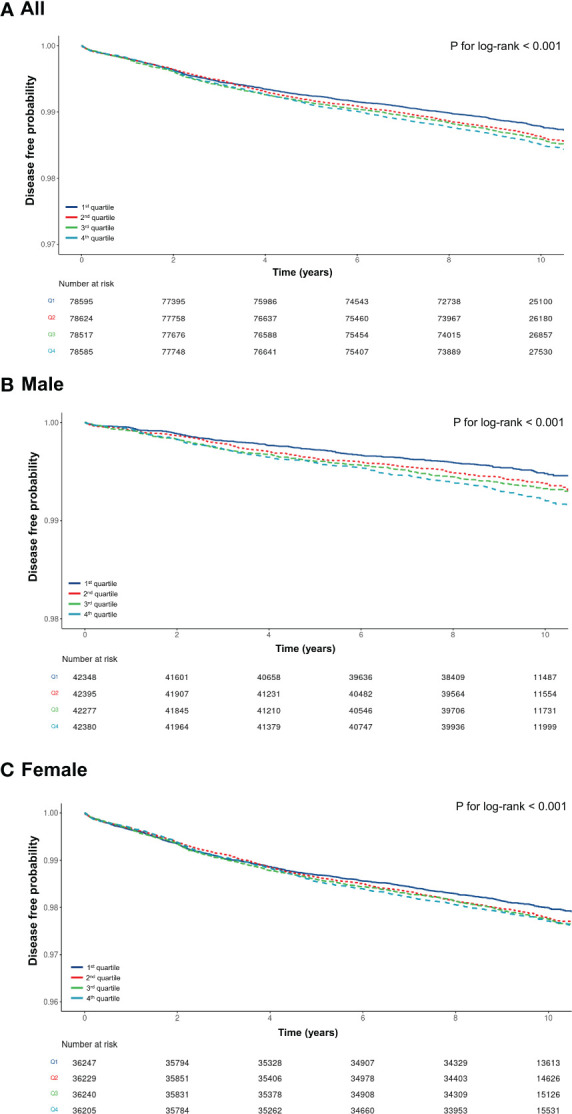
Kaplan-Meier curve for the association between METS-IR values and thyroid cancer.

A total of 4,137 participants (1.3%) were diagnosed with TC during a mean follow-up of 9.5 ± 1.5 years. In the Kaplan-Meier curves, the population with Q1 METS-IR scores showed higher disease-free probabilities than those with Q4 METS-IR scores (**Figure 2A**). However, for women, the curves of the graph were relatively narrower than for men, despite statistically significant differences (*p <*0.001) (**Figures 2B, C**).

The crude and adjusted HRs with 95% CIs for the relationship between METS-IR quartiles and TC risk are described in **Table 2**. In the crude model, the total population and male population in Q2, Q3, and Q4 had significantly increased risks of TC compared to those with Q1 METS-IR values. In women, those in Q2 showed a tendency toward increased TC risk compared to those in Q1, although not statistically significant (*p* = 0.08). However, females in Q3 and Q4 had a statistically increased risk of TC compared with those in Q1 (*p* = 0.03 and 0.01, respectively). In the adjusted model, the total population and female population showed significant differences in TC incidence between Q1 and Q2, Q3, and Q4. In men, there were significant differences in TC incidence between the Q1 population and the Q3 and Q4 populations. However, the TC incidence of those in Q2 was not significantly different compared to those in Q1.

**Table 2:** Hazard ratio and 95% confidence interval for the incidence of thyroid cancer according to METS-IR values.

Subjects (N=314,321)	Events	Follow-up duration (person-years)	Incidence rate (per 1,000 person-years)	Hazard ratio (95% confidence intervals)			
				Crude	P-value	Adjusted*	P-value
Total							
**Q1** **(N=78,595)**	909	738793	1.23	1.00 (reference)		1.00 (reference)	
**Q2** **(N=78,624)**	1033	746928	1.38	1.12 (1.03-1.23)	0.01	1.14 (1.05-1.25)	0.003
**Q3** **(N=78,517)**	1071	745912	1.44	1.16 (1.07-1.27)	0.001	1.21 (1.11-1.33)	< 0.001
**Q4** **(N=78,585)**	1124	746558	1.51	1.22 (1.12-1.33)	< 0.001	1.30 (1.18-1.42)	< 0.001
Male							
**Q1** **(N=42,348)**	200	389602	0.51	1.00 (reference)		1.00 (reference)	
**Q2** **(N=42,395)**	251	394274	0.64	1.24 (1.03-1.49)	0.03	1.17 (0.97-1.41)	0.10
**Q3** **(N=42,277)**	270	397404	0.68	1.33 (1.11-1.60)	0.002	1.23 (1.02-1.48)	0.03
**Q4** **(N=42,380)**	314	398372	0.79	1.54 (1.29-1.84)	< 0.001	1.39 (1.15-1.68)	0.001
Female							
**Q1** **(N=36,247)**	709	344347	2.06	1.00 (reference)		1.00 (reference)	
**Q2** **(N=36,229)**	782	347798	2.25	1.10 (0.99-1.21)	0.08	1.14 (1.03-1.27)	0.01
**Q3** **(N=36,240)**	801	347904	2.30	1.12 (1.01-1.24)	0.03	1.23 (1.11-1.36)	< 0.001
**Q4** **(N=36,205)**	810	347568	2.33	1.14 (1.03-1.26)	0.01	1.30 (1.17-1.45)	< 0.001

*The model was adjusted for age, sex, income level, residence, hypertension, diabetes, dyslipidemia, Charlson comorbidity index, hemoglobin level, glomerular filtration rate, smoking, alcohol drink, and regular exercise status.

**Figure 3:**
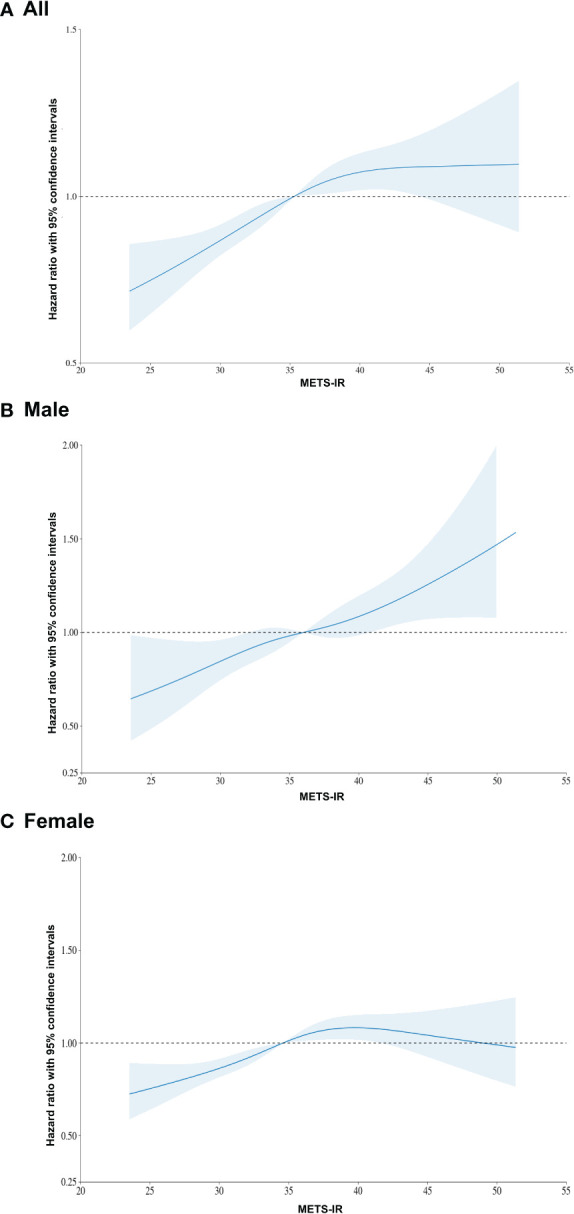
Restricted cubic spline of hazard ratio with 95% confidence intervals for thyroid cancer according to METS-IR values.

The restricted cubic spline, a mathematical function employed to model nonlinear relationships between a continuous predictor variable and an outcome variable, such as adjusted HRs with 95% CIs, is presented in **Figure 3**. The incidence of TC tended to increase with increasing METS-IR values in the total population, especially the male population (**Figures 3A, B**). In women, as METS-IR values increased beyond a certain point, the incidence of TC tended to decrease (**Figure 3C**).

### Subgroup analysis

**Figure 4:**
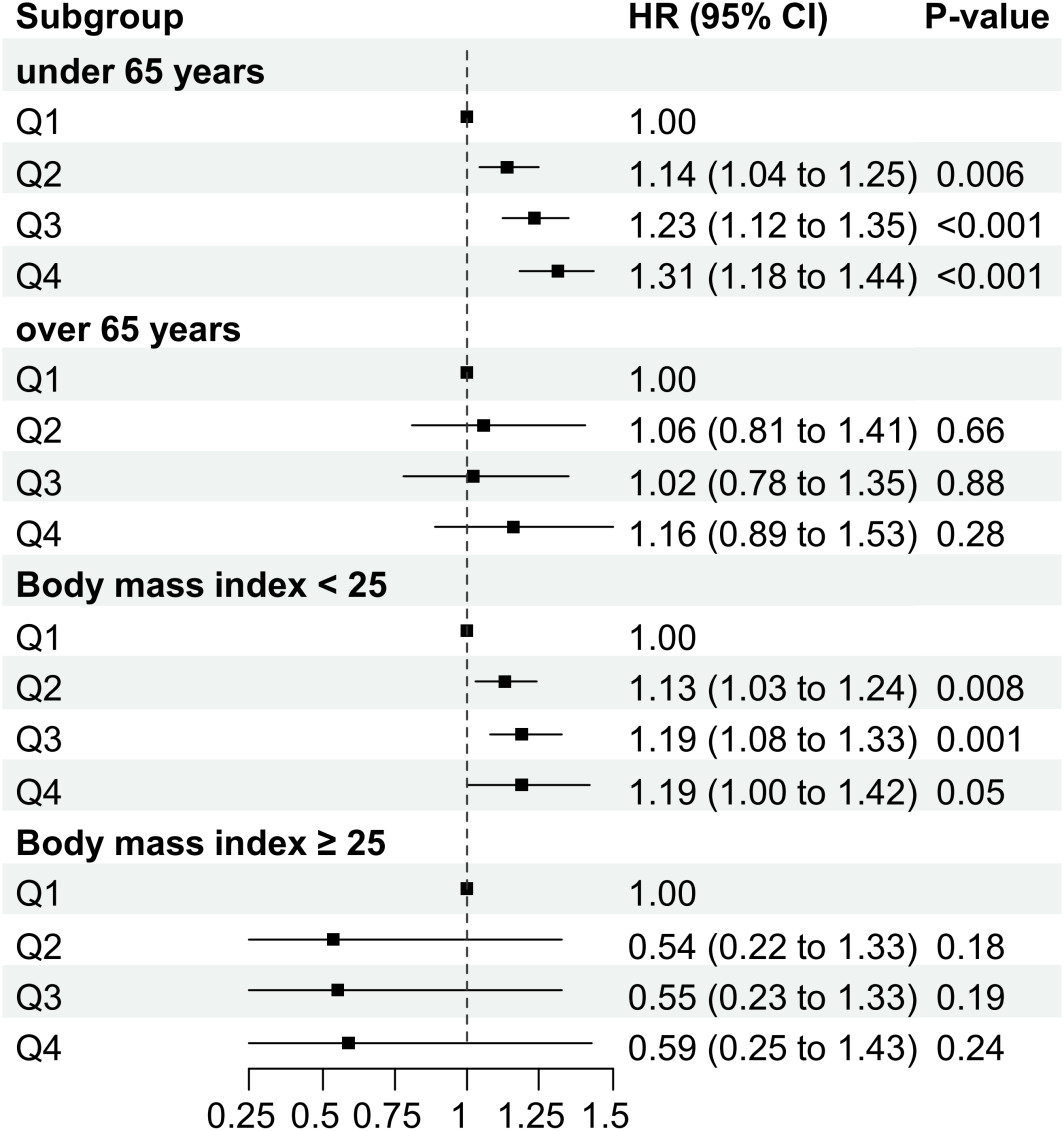
Subgroup analysis according to age and BMI.

Subgroup analysis according to age and BMI is described in **Figure 4**. In the subgroup under 65 years of age, those with Q1 METS-IR scores showed higher disease-free probabilities than those with Q4 METS-IR scores. However, the difference in disease-free probabilities between the Q1 and Q4 populations was not statistically significant. Also, a trend toward increased TC risk was seen in the BMI < 25 kg/m^2^ subgroup with higher METS-IR scores, but this trend was not seen in the BMI ≥ 25 kg/m^2^ subgroup.

### Sensitivity analysis with propensity score matching between Q1 and Q4 groups

Baseline characteristics of the study population before and after propensity score matching were described in **Supplementary Table 4**. After propensity score matching between Q1 and Q4 groups, there were 69,426 subjects with no significant differences in baseline characteristics. After adjustments, the HRs with 95% CIs for TC risk compared Q4 to Q1 were 1.31 (1.15 to 1.49). Regarding sex, the HRs with 95% CIs for TC risk compared Q4 to Q1 were 1.59 (1.19 to 2.13) in males and 1.25 (1.08 to 1.44) in females, respectively.

## Discussion

MetS refers to the presence of a cluster of risk factors specific to cardiovascular disease. According to the National Heart, Lung, and Blood Institute, the cluster of metabolic factors includes abdominal obesity, high blood pressure, impaired fasting blood glucose levels, high TG levels, and low HDL-C levels (23). MetS has been reported to possibly increase the risk of cancers, such as liver, colorectum, and mammary tissue cancer, as well as cardiovascular disease (16). MetS changes the microenvironment of the organism and is accompanied by insulin resistance, both of which are related to tumor formation (19). As with these malignancies, previous studies showed that the incidence of thyroid cancer was associated with MetS (19–21).

Son et al. suggested that obesity was positively correlated with the risk of TC after an average follow-up of seven years in Koreans aged over 20 years (24). Another prospective cohort study found a positive association between BMI and thyroid cancer, especially in women (20). Kown et al. concluded that weight gain significantly increased the incidence of TC, but weight loss was a protective factor for TC (25). Like obesity, high blood pressure and dyslipidemia have been positively linked to TC (21, 26). High FBG levels and diabetes mellitus suggest insulin resistance, but their impact on TC risk is still controversial (19, 27). A prospective cohort study showed that glucose was associated with an increase in TC risk in men. In contrast, an inverse association was seen between glucose and TC risk in women (20). In another matched study, history and the duration of diabetes mellitus were not associated with TC risk, but women with diabetes mellitus for fewer than five years had a reduced risk of TC (27).

In Korea, TC was expected to be the most common form of solid tumor in the 15–34 age group in both sexes, and the incidence of TC is higher in women compared to men (1). In women, the age-specific incidence of TC, along with breast cancer, is expected to level off after the age of 40 years (1). Similar differences in the incidence of TC between the sexes worldwide have been reported (28). In a previous cohort study and meta-analysis, gender disparity was mostly confined to the detection of small subclinical papillary TC (PTC) (29), which has the best prognosis of any subtype of TC. While TC incidence differs between the sexes, the difference in mortality due to TC is not significant (28), which could also be explained by the higher frequency of PTC in women than in men. The reasons for the gender disparity in TC incidence are not fully understood, but female sex hormones possibly play a role (30). Several risk factors for TC, including thyroid-stimulating hormone (TSH), adipokines, and angiotensin II, as well as MetS and estrogen, have been identified (19). A previous study concluded that the aggressiveness of PTC was associated with MetS, which was still significant after adjusting for age, sex, TSH, and BMI (31). However, less is known about the relationship between other thyroid cancer subtypes and MetS.

Given this, a causal relationship between METS-IR and TC incidence would be expected to be stronger in women. As expected, in this study, the female population showed a clearer association between METS-IR values and TC incidence in the adjusted model. However, in the crude model, the relationship was clear in men (**Table 2**). In women, as METS-IR values increased beyond a certain point, the incidence of TC tended to decrease (**Figure 3C**). We suspect that the risk factors used in the adjustment affected the results. Thus, further research is needed to determine which risk factors have an impact, especially if the METS-IR value is above a certain level. Also, the diagnosis of other cancers, such as breast cancer, before TC diagnosis might affect the incidence of TC in the female population with high METS-IR values.

In subgroup analysis, the association between METS-IR scores and TC risk was stronger in younger patients (< 65 years old) and in patients with low BMI (BMI < 25 kg/m^2^). The relationship between METS-IR scores and TC risk was much clearer, with statistical significance in the subgroup < 65 years old. However, in the subgroup ≥ 65 years old, there were no statistically significant differences between METS-IR scores and TC risk (**Figure 4**). Based on this, it is thought that age and BMI play a more important role than other MetS factors in the development of TC, although further study is needed.

This study had some limitations. First, whereas TCs are known as heterogenous in histologic and genetics and have different prognoses according to the subtypes (32), this study could not include information on heterogeneity. Second, information about hormones, such as TSH and estrogen, and other hidden covariates that could affect the incidence of TC was lacking. Also, as this study was retrospective, it utilized only TG, FBG, HDL-C, and BMI measurements at the time of health examination. Thus, we could not assess the extent of changes in these metrics and their potential impact on TC incidence. Despite these limitations, we think that this study is valuable in several ways. To the best of our knowledge, this was the first large cohort study to compare the association between METS-IR and TC incidence. Also, it is thought that this study could be representative of the Korean population because it used nationwide population-based data. Furthermore, we explored the relationship between METS-IR values and TC incidence using various analytic methods.

Despite the high incidence of TC, thyroid ultrasonography is still not included in basic health screening in Korea. One of the reasons for this is concern about overdiagnosis of TC (33), because differentiated TC, including PTC and follicular TC, has good prognosis (34). However, there is a concern that many cases of TC might exist but remain undetected. Also, in the clinic, some advanced TC afflicts many patients and increases socioeconomic costs. Thus, selective screening could be implemented for people at high risk of TC. Also, when the relationship between the aggressiveness of PTC and MetS is additionally considered (31), the results of this study are expected to provide information for which population should be screened for TC. As various targeted therapies have been developed and used in cancer treatment, including TC (35, 36), it would be worthwhile to further investigate the correlation of METS-IR and molecular genetic alterations in TC.

## Conclusion

METS-IR was positively correlated with TC incidence in Korea.

## Data availability statement

The datasets generated during and/or analyzed during the current study are available from the corresponding author on reasonable request. Requests to access these datasets should be directed to Hye Ryeon Kim, hyeryeon13@dau.ac.kr.

## Ethics statement

The studies involving humans were approved by Institutional Review Board of Dong-A University Hospital (DAUHIRB-EXP-23-177). The studies were conducted in accordance with the local legislation and institutional requirements. Written informed consent for participation was not required from the participants or the participants’ legal guardians/next of kin in accordance with the national legislation and institutional requirements.

## Author contributions

HK: Writing – original draft, Writing – review & editing. MS: Conceptualization, Data curation, Formal analysis, Methodology, Writing – original draft, Writing – review & editing. SH: Writing – review & editing. SM: Writing – review & editing. HM: Writing – review & editing. YK: Writing – review & editing. MK: Writing – review & editing. JL: Conceptualization, Writing – review & editing.
