# Supplementary material for: Relationship between METS-IR and thyroid cancer incidence in Korea: a nationwide population-based study
Source: Front Oncol. 2024 Apr 11;14:1383864. doi: 10.3389/fonc.2024.1383864 (PMC11044182; doi:10.3389/fonc.2024.1383864)
Supplement: Supplementary file 1 [file Table_1.docx]

**Supplementary Materials**

**Supplementary Table 1. Definitions of clinical variables**

| **Clinical variables** | **ICD-10 codes + claims codes** | **Health screening** |
| --- | --- | --- |
| Hypertension | I10-I11 + Prescription of ≥ 1 anti-hypertensive drugs | Systolic blood pressure ≥ 140 mmHg or Diastolic blood pressure ≥ 90 mmHg |
| Diabetes | E10-E14 + Prescription of ≥ 1 anti-diabetic drugs | Fasting blood glucose ≥ 126 mg/dL |
| Dyslipidemia | E78 + Prescription of ≥ 1 lipid-lowering drugs | Total cholesterol 240 mg/dL or  LDL cholesterol 190 mg/dL or  Triglycerides 500 mg/dL or  HDL cholesterol < 40 mg/dL |
| Thyroid cancer | C73 |  |

**Supplementary Table 2. Baseline characteristics of the study population according to METS-IR values (males)**

| **All subjects (N=169,400)** | **METS-IR (male)** | | | | **P-value** |
| --- | --- | --- | --- | --- | --- |
|  | **1^st^ quartile, Q1 (N=42,348)** | **2^nd^ quartile, Q2 (N=42,395)** | **3^rd^ quartile, Q3 (N=42,277)** | **4^th^ quartile, Q4 (N=42,380)** |  |
| **Demographics** |  |  |  |  |  |
| Age (years) | 59.4 (9.2) | 58.3 (8.5) | 57.9 (8.2) | 57.4 (7.9) | < 0.001 |
| Income level (%) |  |  |  |  | < 0.001 |
| 1^st^ quartile | 5041 (11.9) | 4634 (10.9) | 4549 (10.8) | 4693 (11.1) |  |
| 2^nd^ quartile | 8791 (20.8) | 7795 (18.4) | 7306 (17.3) | 7247 (17.1) |  |
| 3^rd^ quartile | 12916 (30.5) | 12429 (29.3) | 12332 (29.2) | 12643 (29.8) |  |
| 4^th^ quartile | 15600 (36.8) | 17537 (41.4) | 18090 (42.8) | 17797 (42.0) |  |
| Residence (%) |  |  |  |  | 0.001 |
| Urban | 27779 (65.6) | 28056 (66.2) | 27966 (66.1) | 27462 (64.8) |  |
| Rural | 14569 (34.4) | 14339 (33.8) | 14311 (33.9) | 14918 (35.2) |  |
| **Underlying disease** |  |  |  |  |  |
| Hypertension (%) | 15167 (35.8) | 18778 (44.3) | 21157 (50.0) | 24748 (58.4) | < 0.001 |
| Diabetes (%) | 3377 (8.0) | 5449 (12.9) | 7463 (17.7) | 10429 (24.6) | < 0.001 |
| Dyslipidemia (%) | 8462 (20.0) | 12900 (30.4) | 17267 (40.8) | 24975 (58.9) | < 0.001 |
| Charlson comorbidity index |  |  |  |  | < 0.001 |
| 0 | 23028 (54.4) | 21720 (51.2) | 20682 (48.9) | 19142 (45.2) |  |
| 1 | 10931 (25.8) | 11201 (26.4) | 11263 (26.6) | 11161 (26.3) |  |
| 2 | 4740 (11.2) | 5082 (12.0) | 5328 (12.6) | 5956 (14.1) |  |
| ≥ 3 | 3649 (8.6) | 4392 (10.4) | 5004 (11.8) | 6121 (14.4) |  |
| **Health screening** |  |  |  |  |  |
| Body mass index (kg/m^2^) | 21.1 (1.6) | 23.4 (1.4) | 24.9 (1.5) | 26.9 (1.9) | < 0.001 |
| Systolic blood pressure (mmHg) | 123.6 (15.1) | 125.9 (14.6) | 127.2 (14.2) | 128.9 (14.3) | < 0.001 |
| Diastolic blood pressure (mmHg) | 76.9 (9.7) | 78.4 (9.7) | 79.3 (9.6) | 80.5 (9.7) | < 0.001 |
| Fasting blood glucose (mg/dL) | 96.1 (17.9) | 100.4 (21.4) | 104.0 (24.1) | 109.6 (28.8) | < 0.001 |
| Total cholesterol (mg/dL) | 192.5 (33.9) | 196.1 (35.8) | 197.0 (36.6) | 196.1 (36.9) | < 0.001 |
| Triglycerides (mg/dL) | 98.1 (45.0) | 124.2 (57.1) | 150.8 (68.6) | 192.9 (82.1) | < 0.001 |
| HDL cholesterol (mg/dL) | 61.4 (15.3) | 53.7 (10.5) | 48.8 (9.5) | 43.2 (8.6) | < 0.001 |
| LDL cholesterol (mg/dL) | 111.9 (35.3) | 117.6 (35.5) | 118.2 (36.7) | 114.7 (37.3) | < 0.001 |
| Hemoglobin (g/dL) | 14.3 (1.2) | 14.6 (1.2) | 14.8 (1.2) | 14.9 (1.2) | < 0.001 |
| Glomerular filtration rate (mL/min/1.73 m^2^) | 81.3 (34.1) | 79.1 (32.1) | 78.2 (35.5) | 77.1 (35.8) | < 0.001 |
| Current smoker (%) | 14156 (33.4) | 12047 (28.4) | 11889 (28.1) | 12408 (29.3) | < 0.001 |
| Drink alcohol (%) | 26233 (61.9) | 26811 (63.2) | 26491 (62.7) | 25931 (61.2) | < 0.001 |
| Exercise regularly (%) | 2309 (5.5) | 2436 (5.7) | 2210 (5.2) | 2121 (5.0) | < 0.001 |
| METS-IR | 29.2 (2.1) | 34.0 (1.0) | 37.6 (1.1) | 43.0 (2.7) | < 0.001 |

**Supplementary Table 3. Baseline characteristics of the study population according to METS-IR values (females)**

| **All subjects (N=144,921)** | **METS-IR (female)** | | | | **P-value** |
| --- | --- | --- | --- | --- | --- |
|  | **1^st^ quartile, Q1 (N=36,247)** | **2^nd^ quartile, Q2 (N=36,229)** | **3^rd^ quartile, Q3 (N=36,240)** | **4^th^ quartile, Q4 (N=36,205)** |  |
| **Demographics** |  |  |  |  |  |
| Age (years) | 57.5 (8.9) | 58.8 (8.7) | 60.2 (8.8) | 61.4 (8.8) | < 0.001 |
| Income level (%) |  |  |  |  | < 0.001 |
| 1^st^ quartile | 6246 (17.2) | 6272 (17.3) | 6264 (17.3) | 6255 (17.3) |  |
| 2^nd^ quartile | 8601 (23.7) | 8459 (23.3) | 8297 (22.9) | 8358 (23.1) |  |
| 3^rd^ quartile | 9646 (26.6) | 10381 (28.7) | 10907 (30.1) | 11393 (31.5) |  |
| 4^th^ quartile | 11754 (32.4) | 11117 (30.7) | 10772 (29.7) | 10199 (28.2) |  |
| Residence (%) |  |  |  |  | < 0.001 |
| Urban | 23965 (66.1) | 23016 (63.5) | 22550 (62.2) | 21635 (59.8) |  |
| Rural | 12282 (33.9) | 13213 (36.5) | 13690 (37.8) | 14570 (40.2) |  |
| **Underlying disease** |  |  |  |  |  |
| Hypertension (%) | 10807 (29.8) | 14513 (40.1) | 18220 (50.3) | 23013 (63.6) | < 0.001 |
| Diabetes (%) | 1637 (4.5) | 2821 (7.8) | 4486 (12.4) | 7683 (21.2) | < 0.001 |
| Dyslipidemia (%) | 9926 (27.4) | 12862 (35.5) | 16174 (44.6) | 21167 (58.5) | < 0.001 |
| Charlson comorbidity index |  |  |  |  | < 0.001 |
| 0 | 18374 (50.7) | 16213 (44.8) | 14412 (39.8) | 12096 (33.4) |  |
| 1 | 10178 (28.1) | 10571 (29.2) | 10583 (29.2) | 10300 (28.4) |  |
| 2 | 4528 (12.5) | 5221 (14.4) | 5763 (15.9) | 6273 (17.3) |  |
| ≥ 3 | 3167 (8.7) | 4224 (11.7) | 5482 (15.1) | 7536 (20.8) |  |
| **Health screening** |  |  |  |  |  |
| Body mass index (kg/m^2^) | 20.9 (1.5) | 23.0 (1.4) | 24.7 (1.6) | 27.2 (2.3) | < 0.001 |
| Systolic blood pressure (mmHg) | 119.3 (15.1) | 122.6 (15.2) | 125.4 (15.3) | 128.5 (15.3) | < 0.001 |
| Diastolic blood pressure (mmHg) | 73.8 (9.8) | 75.5 (9.7) | 76.9 (9.8) | 78.5 (9.8) | < 0.001 |
| Fasting blood glucose (mg/dL) | 92.1 (13.9) | 95.3 (16.7) | 98.8 (20.1) | 105.2 (26.8) | < 0.001 |
| Total cholesterol (mg/dL) | 204.7 (35.9) | 205.9 (37.1) | 207.0 (38.6) | 206.5 (39.4) | < 0.001 |
| Triglycerides (mg/dL) | 90.7 (38.5) | 110.2 (47.9) | 132.9 (60.2) | 167.3 (75.1) | < 0.001 |
| HDL cholesterol (mg/dL) | 65.4 (17.1) | 57.9 (11.3) | 53.3 (10.7) | 47.7 (10.2) | < 0.001 |
| LDL cholesterol (mg/dL) | 121.3 (34.6) | 125.8 (35.4) | 127.0 (37.5) | 125.2 (37.7) | < 0.001 |
| Hemoglobin (g/dL) | 12.7 (1.1) | 12.8 (1.1) | 12.9 (1.1) | 13.0 (1.1) | < 0.001 |
| Glomerular filtration rate (mL/min/1.73 m^2^) | 79.3 (25.1) | 78.9 (27.8) | 77.5 (25.6) | 76.5 (26.1) | < 0.001 |
| Current smoker (%) | 637 (1.8) | 526 (1.5) | 522 (1.4) | 569 (1.6) | 0.008 |
| Drink alcohol (%) | 5688 (15.7) | 5215 (14.4) | 4736 (13.1) | 3944 (10.9) | < 0.001 |
| Exercise regularly (%) | 1440 (4.0) | 1393 (3.8) | 1431 (3.9) | 1236 (3.4) | < 0.001 |
| METS-IR | 2.0 ± 0.1 | 2.2 ± 0.1 | 2.3 ± 0.1 | 2.5 ± 0.1 | < 0.001 |

**Supplementary Table 4. Baseline characteristics of the study population before and after propensity score matching between Q1 and Q4 groups.**

| **Variables** | **Before PSM (n = 157,180)** | | | **After PSM (n = 69,426)** | | |
| --- | --- | --- | --- | --- | --- | --- |
|  | **Q1 group (n = 78,595)** | **Q4 group (n = 78,585)** | **P-value** | **Q1 group (n = 34,713)** | **Q4 group (n = 34,713)** | **P-value** |
| **Demographics** |  |  |  |  |  |  |
| Age (years) | 58.5 (9.1) | 59.3 (8.6) | < 0.001 | 58.2 (8.3) | 58.2 (8.3) | 1.00 |
| Sex (%) |  |  | 0.85 |  |  | 1.00 |
| Male | 42348 (53.9) | 42380 (53.9) |  | 16,929 (48.8) | 16,929 |  |
| Female | 36247 (46.1) | 36205 (46.1) |  | 17,784 (51.2) | 17,784 |  |
| Income level (%) |  |  | < 0.001 |  |  | 1.00 |
| 1^st^ quartile | 11287 (14.4) | 10948 (13.9) |  | 4375 (12.6) | 4375 (12.6) |  |
| 2^nd^ quartile | 17392 (22.1) | 15605 (19.9) |  | 6948 (20.0) | 6948 (20.0) |  |
| 3^rd^ quartile | 22562 (28.7) | 24036 (30.6) |  | 10668 (30.7) | 10668 (30.7) |  |
| 4^th^ quartile | 27354 (34.8) | 27996 (35.6) |  | 12722 (36.6) | 12722 (36.6) |  |
| Residence (%) |  |  | < 0.001 |  |  | 1.00 |
| Urban | 51744 (65.8) | 49097 (62.5) |  | 23066 (66.4) | 23066 (66.4) |  |
| Rural | 26851 (34.2) | 29488 (37.5) |  | 11647 (33.6) | 11647 (33.6) |  |
| **Underlying diseases** |  |  |  |  |  |  |
| Hypertension | 25974 (33.0) | 47761 (60.8) | < 0.001 | 15823 (45.6) | 15823 (45.6) | 1.00 |
| Diabetes | 5014 (6.4) | 18112 (23.0) | < 0.001 | 2439 (7.0) | 2439 (7.0) | 1.00 |
| Dyslipidemia | 18388 (23.4) | 46142 (58.7) | < 0.001 | 13229 (38.1) | 13229 (38.1) | 1.00 |
| Charlson comorbidity index |  |  | < 0.001 |  |  | 1.00 |
| 0 | 41402 (52.7) | 31238 (39.8) |  | 17957 (51.7) | 17957 (51.7) |  |
| 1 | 21109 (26.9) | 21461 (27.3) |  | 9506 (27.4) | 9506 (27.4) |  |
| 2 | 9268 (11.8) | 12229 (15.6) |  | 3969 (11.4) | 3969 (11.4) |  |
| ≥ 3 | 6816 (8.7) | 13657 (17.4) |  | 3281 (9.5) | 3281 (9.5) |  |
| **Health screening** |  |  |  |  |  |  |
| Current smoker (%) | 14793 (18.8) | 12977 (16.5) | < 0.001 | 5126 (14.8) | 5126 (14.8) | 1.00 |
| Drink alcohol (%) | 31921 (40.6) | 29875 (38.0) | < 0.001 | 12923 (37.2) | 12923 (37.2) | 1.00 |
| Exercise regularly (%) | 3749 (4.8) | 3357 (4.3) | < 0.001 | 610 (1.8) | 610 (1.8) | 1.00 |
